# Determinants of pneumonia among children attending public health facilities in Worabe town

**DOI:** 10.1038/s41598-022-10194-z

**Published:** 2022-04-13

**Authors:** Roza kumdin Seramo, Shikur Mohammed Awol, Yasin Awol Wabe, Musa Mohammed Ali

**Affiliations:** 1 Silite Zone Health Department, Worabe, Ethiopia; 2 Saint Paul’s Hospital Millennium Medical College, Addis Ababa, Ethiopia; 3 Worabe Comprehensive Specialized Hospital, Worabe, Ethiopia; 4 School of Medical Laboratory Science, College of Medicine and Health Sciences, Hawassa University, Hawasa, Ethiopia

**Keywords:** Immunology, Signs and symptoms

## Abstract

Childhood pneumonia is common in developing countries, with significant morbidity and mortality. Taking the significance of the problem and variability of risk factors into account, a study was needed to identify the potential determinants of pneumonia in under-five children. A facility-based unmatched case–control study was conducted among 435 children (145 cases and 290 controls) aged 2–59 months at public health facilities in Worabe town from December 28, 2016, to January 30, 2017. Data were collected using interviewer-administered questionnaire and analysed using SPSS version 22. Bivariable and multivariable binary logistic regression were used to determine association between dependant and independent variables. Among the factors assessed, stunting [AOR = 3.6,95% CI: 1.9–6.9], carrying the child on the back during cooking [AOR = 2.0,95% CI: 1.2–3.2], absence of chimney in the cooking room [AOR = 2.2, 95% CI: 1.3–3.7], having a history of asthma [AOR = 5.0,95% CI: 2–12], and a previous acute upper respiratory tract infection [AOR = 3.7,95% CI:2.3–6.1] were significantly associated with pneumonia.

## Introduction

Pneumonia is a respiratory tract infection that affects children. *Streptococcus pneumoniae*, *Haemophilus influenzae* type b (Hib), and respiratory syncytial virus are among the common pathogens involved^1^. *S. pneumoniae* is the most common cause of bacterial pneumonia during childhood in the developing countries. Microbes that are commonly found in upper respiratory tract can cause lower respiratory tract infection. They may also spread via air-borne droplets at the time of cough or sneeze or may spread through blood, especially during and shortly after birth^2^. Pneumonia is the major killer of under-five children than any other disease known to affect children. Childhood pneumonia causes substantial mortality and morbidity among under-five children, with developing nations carrying the highest pneumonia burden^3,4^.

Every year, nearly 1 million children die of pneumonia worldwide and approximately about 15% of all deaths occur in children under age of five. This means a loss of over 2500 children’s lives every day or 100 children every hour. It is the most prevalent in sub-Saharan Africa and South Asia, Nigeria, Pakistan, the Democratic Republic of Congo, and Ethiopia, accounting for 50% of total deaths^5–7^. In Ethiopia, pneumonia is the leading cause of death, accounting for 18% of all under-five mortality burdens^8^. As a result, Ethiopia ranks fifth (62 deaths in 1000) among 15 countries in the world and has the highest death rate from clinical pneumonia in under five children^9^. It also causes millions of morbidity and adds costs to the health services program; as health services are passed on to cure high pneumonia morbidity cases, with an estimated 3,370,000 children encountering pneumonia annually^10,11^.

Ethiopia follows the same strategy and has adopted the World Health Organization (WHO) Integrated Management of Childhood Illness (IMNCI) guidelines since 2001. Moreover, with the objective of increasing access to life-saving interventions, Ethiopia made a policy breakthrough of introducing community-based treatment of pneumonia through health extension workers in 2010^12^. Since then, 38,000 health extension workers from nearly 15,000 health posts have been equipped with the skills and supplies to treat pneumonia at the community level using the integrated community case management (ICCM) approach^13^.

Previous studies indicated that some of most determinants for pneumonia include the educational status of parents, age of the mother, family child caring practice, family income, age and sex of the child, parental smoking or indoor air pollution from biomass fuel smoke, absence window in the kitchen, overcrowding, parental asthma, household history of pneumonia, malnutrition, lack exclusively breastfeeding, lack of zinc supplementation, comorbidity condition such as diarrhoea, measles, acute upper respiratory infection (AURTI), and previous history of asthma^1,10,14–37^.

Contributing factors of pneumonia need to be studied to better inform health care providers and policy-makers to consider pneumonia prevention strategies. Although there are studies conducted in Ethiopia, the risk factors for pneumonia differ in different societies and study populations^37^. Taking the high burden of pneumonia and variability of risk factors into account, this study is envisaged to identify the potential determinants of pneumonia in under-five children. Above all, there was no previous study in the study area that could show the determinants of pneumonia.

## Methods

### Study setting and study design

An unmatched case–control study was conducted from December 28, 2016, to January 30, 2017, in public health facilities located in Worabe town, Silte zone, Southern Nation Nationalities, and People’s Region. Worabe town is the capital town of Silte zone. The town has a total population of 29,600, of which 4618 are under-five children. There are two public health facilities in Worabe town and 6 private clinics^38^.

#### Study population

Children who were 2–59 months old, and those who visited the selected health facilities during the study period.

Cases were children aged 2–59 months who visited pediatric units, registered and diagnosed with pneumonia as defined by the Federal Democratic Republic of Ethiopian Ministry of Health Integrated Management of Childhood Illness (IMNCI) guideline that is adapted from WHO^39^.

The control group was defined as children aged 2–59 months without pneumonia and attended the public health facilities in Worabe town.

### Sample size determination and sampling technique

A sample size of 435(145 cases and 290 controls) was determined using Epi-Info version 3.5.4 statistical software assumingtwo side confidence level (Cl) of 95%, power = 80%, ratio control to case 2:1, and taking a history of AURTI as a predictor of pneumonia with 22.4% prevalence among control group; 1.80 Odds ratio from a case–control study^21^ and an estimated non-response rate of 10%. All public health facilities in the town were purposively included based on patient load and the presence of accessible trained staff on IMNCI. The sample size was distributed to each health facility based on the average daily caseload. According to the zonal health bureau Health Management Information System report, the average daily pneumonia patients among under-five children at Worabe comprehensive specialized hospital (WCSH) was seven and it was two at Worabe Health Centre^40^. Based on this, the sample size allocated for the Worabe health centre was one case and two controls on a daily basis with a total of 21 cases and 42 controls. The sample size allocated for WCSH was six cases and twelve controls on a daily basis with a total of 124 cases and 248 controls.

#### Selection of cases

All cases (diagnosed and recorded as pneumonia/severe pneumonia) were considered in the study until the required sample size was reached/fulfilled.

#### Selection of controls

As the control-to-case ratio was 2:1, two children who did not have pneumonia and visited selected health facilities for different services at the time of data collection were randomly selected by systematic random sampling after the cases were identified.

### Eligibility criteria

The study included children who were between 2 and 59 months of age, those who were residents of Worabe town for a minimum of six months, and visited the pediatric unit of WCSH and Worabe Health Center during the study period.

Children with the following conditions were excluded from the study: cardiac disease, cough that lasted for > 15 days (suspected of pulmonary tuberculosis), cough because of the recent history of aspiration of a liquid or a foreign body, and caregiver who did not have any information about the child at the time of data collection.

### Study variables

#### Dependent variables

Presence of pneumonia.

#### Independent variables

Socio-demographic factors: Parental factors such as educational and occupational status, parental cigarette smoking, age of the mother, family size, and family caring practice (parental/home maid, place of child during cooking, and family income).

Child factors: age and sex, immunization status, a pre-existing illness such as a history of diarrhoea, AURTI, and acute lower respiratory tract infection/pneumonia in the last 2 weeks and asthma.

Environmental factors: type of fuel used for cooking, crowding status, place of cooking, parental asthma, and household history of tuberculosis and pneumonia.

Nutritional condition of the child: undernutrition, breastfeeding status of the child for the first 6 months and duration of breastfeeding, age of complementary feeding started, and zinc supplementation.

### Operational definitions

#### Pneumonia

A child aged 2–59 months with cough and/or difficulty in breathing for less than two weeks of duration plus fast breathing and/or chest in drawing^15–19^.

Fast breathing is defined as:

- For children in the age interval of 2–11 months, 50 breaths per minute or more
- For children in the age interval of 12 months to 5 years, 40 breaths per minute or more^39–41^.

History of acute upper respiratory tract infection(AURTI): a child who had a history of ear infection, common cold, tonsillitis, or pharyngitis in the last fifteen days prior to data collection^42^.

Underweight: Weight at the birth of less than 2500 g^43,44^.

Stunting: Chronic undernutrition condition in which a child is short for his or her age^44^.

Wasting: Unintended loss of weight which makes children too thin and weak^45^.

### Data collection tools and procedures

A structured questionnaire was developed based on a review of previously published studies and adapted for the current study with certain modifications^17–28^. The questionnaires included information on the possible risk factors for pneumonia, including socio-demographic factors, environmental/home-based factors, nutritional factors, immunization status, pre-existing illness and child care practices. Data were collected by IMNCI-trained nurses working in under-five clinics who received two days of training regarding the research. After the study participants were identified as cases and controls, mothers/primary caretakers were interviewed based on the interviewer-administered pretested structured questionnaire.

#### Anthropometric measurements

The weight and height of the child were taken at the beginning of the interview by data collectors. A suspended scale of 25 kg capacity graduated at 0.1 kg was used for weighing infants and children. The reading was recorded to the nearest 0.1 kg. Length measurements in the lying position were taken for children less than two years of age, and height measurements were taken for children 2–5 years of age. The anthropometric data were analysed in terms of weight for age, length for age, and weight for length using WHO Anthrosoftware to prepare for SPSS. The WHO (2006) growth standard was used to report anthropometric measurements result by Z-score, and the global acute malnutrition standard was used to classify the child’s nutritional status as normal, stunted, wasted, or underweight.

### Data quality management

The questionnaire was pretested on a 5% sample size at Kbit Primary Hospital to ensure the validity and reliability of the survey tools. After collecting the pre-test data, it was checked for potential problems related to the tool, such as any difficult question that was understandable or unclear to reply and corrective measures were taken.

### Data processing and analysis

The collected data were checked for completeness, coded and entered into Epi Info version 7 and exported to the statistical package for social sciences (SPSS) version 22 for analysis. The entered data were cleaned and checked for consistency and extent of outliers. Different statistical assumptions and appropriate corrections were made prior to analysis. Descriptive analyses were carried out for each of the independent variables. Bivariable and multivariable binary logistic regression analysis was used to test the association between the independent and dependent variables. Bivariable analysis was performed for each of the independent variables with the outcome variable. Variables that had a *p*-value < 0.2 on bivariate analysis were taken as candidates for multivariable binary logistic regression model analysis to identify predictors of the outcome variables. Variables with a *p*-value less than 0.05 on multivariable logistic regression analysis were considered statically significant factors for the outcome variables. The strength of the association between the dependent variable and independent variables was expressed using adjusted odds ratio (AOR) with 95% confidence intervals.

### Ethical approval and consent to participant

The study was ethically approved by the Institutional Review Board of Saint Paul’s Millennium Medical College Department of Public Health. An official permission letter was obtained from the study site. The objectives, expected outcomes, benefits, and risks of the study were explained to mothers/guardians/caregivers of the study participants. Data were collected after written informed consent was obtained. The study was conducted in accordance with the Declaration of Helsinki.

## Results

### Socio-demographic characteristics

A total of 435 (145 cases and 290 controls) children aged 2–59 months participated in the study, making the response rate 100% for both groups. The highest proportion of cases (57.2%) and controls (62.4%) were in the age group of 2–11 months, with mean ages of 14.6(SD ± 12.8) and 12.6 months (SD ± 12.7) for cases and controls respectively. Approximately 52% of cases and 51% of controls were males (Table 1).

**Table 1:** Socio-demographic characteristics of children aged 2–59 months attending public health facilities in Worabe town, Silte zone, 2017(N = 145 cases and 290 controls).

Variables	Cases		Controls		Total		*P*-value	COR(95% C.I)
	Frequency	Percent	Frequency	Percent	Frequency	Percent		
Age of the child								
2–11 months	83	57.2	181	62.4	264	60.7%	0.200	0.7 [0.4, 1.2]
12-23 months	31	21.4	61	21	92	21.1%	0.500	0.8 [0.4, 1.5]
24-59 months	31	21.4	48	16.6	79	18.2%		1
Sex of the child								
Male	76	52.4	148	51	224	51	0.800	1.2 (0.7, 1.6)
Female	69	47.6	142	49	211	49		1
Age of the mother								
18–24	38	26.2	95	32.8	133	31	0.5	0.8 (0.5, 1.5)
25–31	79	54.5	138	47.6	217	50	0.6	1.2 (0.7, 1.9)
≥ 32	28	19.8	57	19.7	85	19		1
Educational status of mother								
Not formal education	51	35.2	80	27.6	131	30.1	0.7	1 (0.6, 2.2)
Primary school	51	35.2	130	44.8	181	41.6	0.29	0.7 (0.4, 1.3)
Secondary school	24	16.6	46	15.9	70	16	0.86	0.9 (0.4, 1.9)
Higher education	19	13.1	34	11.7	53	12.2		1
Educational status of father								
No formal education	44	30.3	71	24.5	115	26.4	0.4	1.3 (0.7, 2.4)
Primary	53	36.6	121	41.7	174	40	0.8	0.9 (0.5, 1.6)
Secondary	21	14.5	41	14.1	62	14.3	0.8	1 (0.5, 2.2)
Higher	27	18.6	57	19.6	84	19.3		1
Mother’s occupation								
House wife	88	60.7	204	70.3	292	67.2	0.2	0.5 (0.2, 1.4)
Civil servant	17	11.7	33	11.4	50	11.5	0.4	0.6 (0.2, 1.9)
Merchant	33	22.8	45	15.5	78	17.9	0.7	0.8 (0.3, 2.5)
Others	7	4.8	8	2.8	15	3.4		1
Father's occupation								
Merchant	51	35.2	124	42.8	175	40.3	0.9	0.9 (0.5, 1.9)
Daily labourers	44	30.3	71	24.4	115	26.4	0.3	1.5 (0.7, 2.9)
Civil servant	31	21.4	50	17.2	81	18.6	0.3	1.5 (0.7, 2.9)
Others	19	13.1	45	15.5	64	14.7		1
Family monthly income in Ethiopian birr								
< 1000	23	15.9	64	22.1	87	20	0.4	0.8 (0.4, 1.4)
1000–1500	27	18.6	39	13.4	66	15.2	0.2	1.4 (0.8, 2.7)
1600–2500	53	36.6	99	34.1	152	34.9	0.7	1.1 (0.7, 1.8)
> 2500	42	29	88	30.3	130	29.9		1
Family size								
2–3	26	17.9	69	23.8	95	21.8	0.1*	0.8 (0.5, 1.3)
4–5	67	46.2	135	46.6	202	46.5	0.4	0.6 (0.4, 1.1)
> 6	52	35.9	86	29.7	138	31.7		1
Family child care practice								
Parental	116	80	228	78.6	344	79	0.7	1 (0.7, 1.8)
Home maid	29	20	62	21.4	91	21		1

*COR* crude odds ratio, *CI* confidence interval, *1* reference group.

### Nutritional, pre-existing illness and vaccination status of the children

The majority (79.3%) of cases and 85.5% of controls were exclusively breastfed for the 1st six months of life. Regarding the nutritional status of the children, 11.9% cases and 9% controls, 26.9% cases and 18.6% controls were underweight and stunted respectively. More than 90% of cases and 95% of controls received pentavalent and pneumococcal conjugate vaccines.

The study indicated that; variables such as Zinc supplementation (*p* = 0.01), stunting (*p* = 0.001), wasting (*p* = 0.06), exclusive breastfeeding for the first 6 months (*p* = 0.1), a child with the previous history of AURTI (*p* < 0.001), and diarrhoea (*p* = 0.06) in the past two weeks and history of asthma (*p* < 0.001) were associated with the occurrence of pneumonia in the bivariate logistic regression at *p* < 0.2 (Table 2).

**Table 2:** Nutritional status, vaccination status and pre-existing illness among children aged 2–59 months attending public health facilities in Worabe town, Silte zone, 2017 (N = 145 cases and 290 controls).

Variables	Case		Control		Total		*P*-value	COR(95% C.I)
	Frequency	Percent	Frequency	Percent	Frequency	Percent		
Breast feeding status of the child during the first 6 months								
Exclusive BF	115	79.3	247	85.2	362	83.2	0.1*	1.2 (0.9, 1.6)
Mixed feeding	30	20.7	43	14.8	73	16.8		1
Zinc Supplementation								
Supplemented	56	38.6	77	26.6	133	30.6	0.01*	1.7 [1.1, 2.7]
Not supplemented	89	61.4	213	73.4	302	69.4		1
Weight for age								
Underweight	17	11.7	26	9	43	9.9	0.4	1.3 (0.7, 2.6)
Normal	128	88.3	264	91	392	90.1		1
Height for age								
Stunted	36	24.8	23	7.9	59	13.6	0.00*	3.8 (2.2, 6.8)
Not stunted	109	75.2	267	92.1	376	86.4		1
Weight for height								
Wasted	29	20	38	13.1	67	15.4	0.06*	1.7 (0.9, 2.8)
Not wasted	116	80	252	86.9	368	84.6		1
Dose the child received Pentavalents vaccine								
Yes	134	92.4	277	95.5	411	94.5	0.2	0.6 (0.3, 1.3)
No	11	7.6	13	4.5	24	5.5		1
Get measles vaccine								
Yes	76	52.4	128	44.1	203	46.7	0.70	1.5 (0.9, 2.3)
No	13	9	28	9.7	50	11.5	0.50	1.3 (0.7, 2.5)
N/A(age < 9 months)	56	38.6	134	46.2	182	41.8		1
Diarrhoea in last 2 weeks								
Yes	44	30.3	54	18.6	98	22.5	0.06*	1.9 [1.2, 3]
No	101	69.7	236	81.4	337	77.5		1
AURTI in the last 2 weeks								
Yes	68	46.9	51	17.6	119	27.4	0.00*	4 [2.7, 6.5]
No	77	53.1	239	82.4	316	72.6		1
Previous history of asthma in the child								
Yes	23	15.9	10	3.4	33	7.6	0.00*	5.3 [2.4, 11.4]
No	122	84.1	280	96.6	402	92.4		1
Pneumonia among family in last 2 weeks								
Yes	34	23.4	61	21	95	21.8	0.6	1.2 (0.7, 1.9)
No	111	76.6	229	79	340	78.2		1
Parental asthma								
Yes	7	4.8	13	4.5	20	4.6	0.8	1.1 (0.4, 2.8)
No	138	95.2	277	95.5	415	95.4		1

*BF* breast feeding, *COR* crude odds ratio, *CI* confidence interval, *1* reference group.

### Environmental factors associated with pneumonia

More than half of the cases (57.2%) and controls (54.8%) lived in households that used wood as a source of energy for cooking. The highest proportion of cases (63.4%) and controls (63.8%) had mothers cooking their food in the kitchen. Approximately 61% of cases and 44% of controls stayed near or on their mothers'/care takers back while she was cooking. The bivariate logistic regression analysis revealed that crowding (*p* = 0.04), type of energy source for cooking (*p* = 0.02), absence of chimneys in the cooking room (*p* < 0.01), and place of a child during cooking (*p* < 0.01) had significant associations with pneumonia among children (Table 3).

**Table 3:** Environmental characteristics of children aged 2–59 months attending public health facilities in Worabe town, Silte zone, 2017 (N = 145 cases and 290 controls).

Variables	Case		Control		Total		*P*-value	COR(95% C.I)
	Frequency	Percent	Frequency	percent	frequency	percent		
Common source of energy used for cooking used the family								
Wood	83	57.2	159	54.8	242	55.6	0.40	1.4 (0.8, 2.3)
Charcoal	21	14.5	41	14.1	62	14.2	0.20	1.4 (0.7, 2.6)
Kerosene	10	6.9	8	2.8	18	4.1	0.02*	3.3 (1.2, 9.1)
Electricity	31	21.4	82	28.3	113	26		1
Common place of cooking used by the family								
In living room	53	36.6	105	36.2	158	36.3	0.70	1.1 (0.7, 1.8)
In kitchen attached to the living room	37	25.5	64	22.1	101	23.2	0.40	1.3 (0.8, 2.1)
In separate kitchen	55	37.9	121	41.7	176	40.5		1
Chimney in the cooking room								
No	101	69.7	140	48.3	241	55.4	< 0.01*	2.5 (1.6, 3.7)
Yes	44	30.3	150	51.7	194	44.6		1
Air flow in the living room								
Yes	124	85.5	261	90	385	88.5	0.20	1.5 (0.8, 2.8)
No	21	14.5	29	10	50	11.5		1
Place of the child during cooking(usual location)								
Near or carried on back	93	64.1	127	43.8	220	50.6	< 0.01*	2.3 [1.5, 3.5]
Outside of cooking room	52	35.9	163	56.2	215	49.4		1
Cigarette smoker among household residents								
Yes	11	7.6	19	6.6	30	6.8	0.70	1.2 (0.5, 2.5)
No	134	92.4	271	93.4	405	93.2		
Crowding status								
Crowded	60	41.4	91	31.4	151	35	0.04*	1.5 [1.1, 2.3]
Not crowded	85	58.6	199	68.6	284	65		1

*COR* crude odds ratio, *CI* confidence interval, *1* reference group.

### Factors associated with pneumonia among children aged 2–59 months

All variables analysed by bivariable binary logistic regression showed a *p*-value less than 0.2. Multivariable binary logistic regression analysis showed that stunting, previous history of asthma diagnosis in the child, place of the child during cooking, absence of chimney in the cooking room and history of AURTI in the last 2 weeks prior to data collection were determinants of pneumonia. The findings revealed that the odds of stunting were 3.6 times higher among children with pneumonia than controls (AOR = 3.6, 95% CI: 1.8, 7; *p* < 0.001). On the other hand,the odds of carrying the child on the back while cooking/beside the cooking mother were twice as high as those of compared to children outside of the cooking room among cases than controls (AOR = 2, 95% CI: 1.2, 3.3; *p* = 0.006).

Similarly, the odds of children living in households with no chimney in the cooking room were 2.2 times higher among cases than controls (AOR = 2, 95% CI: 1.3, 3.7; *p* = 0.003). The odds of having a previous history of asthma were fivefold higher among cases than controls (AOR = 5, 95% CI: 2, 12; *p* < 0.001). Likewise, the odds of having a history of AURIT in the last 2 weeks prior to data collection were 3.7 times higher among children with pneumonia than among children without pneumonia (AOR = 3.7, 95% CI: 2.3, 6.1; *p* < 0.001) (Table 4).

**Table 4:** Determinants of pneumonia among children aged 2–59 months in public health facilities in Worabe town, Southern Ethiopia, 2017(N = 145 cases and 290 controls).

Variables	Cases No (%)	Controls No (%)	COR (95% C.I.)	AOR (95% C.I.)	P-value
Breast feeding status of the child during the first 6 months					
Exclusive BF	115 (79.3%)	247 (85.2%)	1.5 (0.9, 1.6)	0.8 (0.4, 1.5)	0.4
Mixed feeding	30 (20.7%)	43 (14.8%)	1.0	1	
Zinc supplementation					
Supplemented	56 (38.6%)	77 (26.6%)	1.7 [1.2, 2.7]	1.4 (0.8, 2.3)	0.2
Not supplemented	89 (61.4%)	213 (73.4%)	1.0	1	
Height for age					
Stunted	36 (24.8%)	23 (7.9%)	3.8 [2.2, 6.8]	3.6 [1.8, 7]*	< 0.001
Not stunted	109 (75.2%)	267 (92.1%)	1.0	1	
Crowding status					
Crowded	60 (41.4%)	91 (31.4%)	1.5 [1.1, 2.3]	1.2 (0.7, 2)	0.6
Not crowded	85 (58.6%)	199 (68.6%)	1.0		
Place of child during cooking					
Near or carried on back	93 (64.1%)	127 (43.8%)	2.3 [1.5, 3.5]	2.0 [1.2, 3.3]*	0.006
Outside of cooking room	52 (35.9%)	163 (56.2%)	1.0	1	
Type of cooking fuel mostly used the family					
Wood	83 (57.2%)	159 (54.8%)	1.4 (0.8, 2.3)	1.3 (0.7, 2.4)	0.500
Charcoal	21 (14.5%)	41 (14.1%)	1.4 (0.7, 2.6)	0.9 (0.4, 1.9)	0.800
Kerosene	10 (6.9%)	8 (2.8%)	3.3 (1.2, 9.1)	2.3 (0.7, 7.3)	0.200
Electricity	31 (21.4%)	82 (28.3%)	1.0	1	
Weight for height					
Wasted	29 (20%)	38 (13.1%)	1.7 (0.9, 2.8)	1.4 (0.7, 2.7	0.300
Not wasted	116 (80%)	252 (86.9%)	1.0	1	
Family size					
2–3	26 (17.9%)	69 (23.8%)	0.6 (0.4, 1.1)	0.7 (0.3, 1.6)	0.400
4–5	67 (46.2%)	135 (46.6%)	0.8 (0.5, 1.3)	0.9 (0.5, 1.6)	0.700
6and above	52 (35.9%)	86 (29.7%)	1.0	1	
Chimney in the cooking room					
No	101 (69.7%)	140 (48.3%)	2.5 (1.6, 3.7)	2.2 [1.3, 3.7]	0.003*
Yes	44 (30.3%)	150 (51.7%)	1.0	1	
History of diarrhoea in the last 2 weeks					
Yes	44 (33.3%)	54 (18.6%)	1.9 [1.2, 3]	1.5 (0.9, 2.7)	0.1
No	101 (69.7%)	236 (81.4%)	1.0	1	
History of AURTI in the last 2 weeks					
Yes	68 (46.9%)	51 (17.6%)	4 [2.7, 6.5]	3.7 [2.3, 6.1]	< 0.001*
No	77 (53.1%)	239 (82.4%)	1.0	1	
History of asthma in the child					
Yes	23 (15.9%)	10 (3.4%)	5.3 [2.4, 11.4]	5.0 [2, 12]	< 0.001*
No	122 (84.1%)	280 (96.6%)	1.0	1	

*COR* crude odds ratio, *AOR* adjusted odds ratio, *CI* confidence interval, *1* reference group.

## Discussion

In this study, pre-existing respiratory illnesses, such as a history of asthma in the child and a previous history of AURTI, undernutrition (stunting), absence of chimney in the cooking room, and carrying the child on the back during cooking, were found to be significant risk factors associated with the occurrence of pneumonia among children aged 2–59 months.

The odds of carrying the child on the back while cooking was 2 times greater than that of children who pass outside of the cooking room among cases than controls. This could be due to indoor pollution as a result of unsafe energy sources such as charcoal, wood, and biomass. This result is consistent with a cross-sectional study conducted in northwest Ethiopia found that a child who was carried on the back of mother during cooking was five times more likely to develop pneumonia than their counterparts^17^. Similar findings were obtained in different countries that reported indoor air pollution as a risk factor for pneumonia^15,25,28–30^. However, two studies conducted in Ethiopia did not report a significant association between the place of the child during cooking and the occurrence of pneumonia in children^18,23^. The variation of the results could be due to differences in study setting and methodology used.

Stunting was another factor identified to be significantly associated with pneumonia, indicating that the odds of stunting were 3.6 times higher among children with pneumonia than controls, which is in line with a study conducted in Northwest Ethiopia and Bangladesh^17,19^. The possible reason might be that stunting shows long-term malnutrition, which weakens the child’s immunity and makes the child vulnerable to pneumonia. From different prospects of different studies, malnutrition (undernutrition) weakens the respiratory muscles needed to clear secretions in the respiratory tract, which intern predisposes to pneumonia^1,24,41^.

In this study, there was the strongest association between pneumonia and a history of asthma, indicating that children who had a previous history of asthma had approximately fivefold increased odds of developing pneumonia compared to their counterparts. According to the WHO 2008 bulletin report, concomitant diseases such as asthma were likely to be the risk factors for the occurrence of childhood pneumonia^15^. Several findings from other areas also indicated the relationship between asthma and pneumonia episodes^21,36^. In a study conducted in the Philippines, 55.4% of children who developed consolidated pneumonia had asthma as an underlying illness^27^. In the current study, we are unable to conclude whether asthma or other asthmatic condition was a predisposing factor for pneumonia. Further study is needed to define a causal relationship between asthma and pneumonia.

A history of AURTI in a child in the last two weeks preceding the current pneumonia was identified to put a child 3.7 times at risk of pneumonia compared to their counterpart. This is in line with the study conducted in southwest Ethiopia, which indicates that the risk of pneumonia is more than 5 times for children who had previous AURTIs^23^, and a similar finding in the Netherlands showed a strong relationship between the occurrences of community- acquired pneumonia and an increasing number of previous AURTIs^21^. However, a study conducted in Ethiopia reported no significant association between occurrences of pneumonia and preceding infection of AURTI in children^22^. A possible explanation might be that AURTI increases susceptibility to infections that subsequently predisposes patients to pneumonia or that there might be descending infection from the upper to lower respiratory tract.

### Limitations of the study

The diagnosis of pneumonia was based on the clinical WHO IMNCI classification guidelines, which introduced misclassification recall bias; the study did not consider privates health facilities and did not consider the complete population.

## Conclusions

This study indicated stunting, a previous history of asthma and upper respiratory tract infection, the absence of chimney in the cooking room and carrying the child on the back during cooking are strongly associated with an increased risk of childhood pneumonia. Therefore, the zonal health bureau, in collaboration with the agricultural office needs to improve the nutritional status of children, and the town administrative health office, in collaboration with the health facilities may provide health education to the community about the health risk of child exposure to biomass fuel smoke and to have chimneys in the cooking room.

## Data Availability

All necessary raw data used for analysis in this study is available from corresponding author with reasonable request.

## Abbreviations

AURTI: Acute upper respiratory tract infection
ICCM: Integrated community case management
IMNCI: Integrated management of neonatal and childhood illness
WCSH: Worabe comprehensive specialized hospital
WHO: World Health Organization
